# Alternative models for two crystal structures of bovine rhodopsin

**DOI:** 10.1107/S0907444908017162

**Published:** 2008-07-17

**Authors:** Ronald E. Stenkamp

**Affiliations:** a Departments of Biological Structure and Biochemistry, Biomolecular Structure Center, University of Washington, Seattle, WA 98195, USA

**Keywords:** alternate space groups, rhodopsin, G protein-coupled receptors, integral membrane proteins

## Abstract

The space-group symmetry of two crystal forms of rhodopsin (PDB codes 1gzm and 2j4y; space group *P*3_1_) can be re-interpreted as hexagonal (space group *P*6_4_). Two molecules of the G protein-coupled receptor are present in the asymmetric unit in the trigonal models. However, the noncrystallographic twofold axes parallel to the *c* axis can be treated as crystallographic symmetry operations in the hexagonal space group. This halves the asymmetric unit and makes all of the protein molecules equivalent in these structures. Corrections for merohedral twinning were also applied in the refinement in the higher symmetry space group for one of the structures (2j4y).

## Abstract 2

Two crystal structures of rhodopsin that were originally described using trigonal symmetry can be interpreted in a hexagonal unit cell with a smaller asymmetric unit.

Owing to their importance in many signal transduction pathways, G protein-coupled receptors (GPCRs) are target molecules for important therapeutic compounds. Rhodopsin, one of the visual pigments in the retina, was the first GPCR to have its crystal structure determined (Palczewski *et al.*, 2000 ▶). Multiple trigonal and rhombohedral crystal forms have been reported for rhodopsin (Edwards *et al.*, 2004 ▶; Li *et al.*, 2004 ▶; Standfuss *et al.*, 2007 ▶; Salom, Le Trong *et al.*, 2006 ▶; Salom, Lodowski *et al.*, 2006 ▶; Lodowski *et al.*, 2007 ▶).

The structures of ground-state bovine rhodopsin and of one of its recombinant mutants (N2C/D282C) have been solved in space group *P*3_1_ (Edwards *et al.*, 2004 ▶; Li *et al.*, 2004 ▶; Standfuss *et al.*, 2007 ▶) with two molecules in the asymmetric unit. The two molecules (PDB entries 1gzm and 2j4y) have similar crystal structures, but they are significantly non-isomorphous (*a* = *b* = 103.8, *c* = 76.6 Å for the ground state, *a* = *b* = 109.3, *c* = 77.7 Å for the mutant). A major intermolecular interaction important for crystal packing involves two antiparallel α-helices from different molecules. These helix–helix interactions differ in the two crystal structures by a ‘sliding’ translocation along the helical axes. While investigating the nature of this non-isomorphism, it became apparent that the crystal structures could also be described in space group *P*6_4_ with one molecule in the asymmetric unit.

In the original crystallographic analysis of the ground-state molecule, the choice of space-group symmetry, *i.e.* trigonal *versus* hexagonal, was made mostly on the basis of a reduced κ = 60° peak in the native rotation function. *R*_merge_ values were not significantly different for the two choices of Laue symmetry. The two molecules in the asymmetric unit of the trigonal unit cell are related by a noncrystallographic twofold rotation axis parallel to the 3_1_ screw axis. After the structure was solved, the original authors revisited the space-group assignment. The possibility of the structure being in space group *P*6_2_ was considered and rejected. No consideration of *P*6_4_ is described in the original structure reports. Because the symmetry operations in *P*3_1_ are a subset of those in *P*6_4_, the crystal structures of both rhodopsins can be successfully refined in that space group.

Refinement of the ground-state structure started with the coordinates and reflection data deposited with PDB code 1gzm. *R*_merge_ for the reflections related by the hexagonal symmetry was 0.043. Averaging them reduced the number of unique reflections to 13 785. The *R*_merge_ reported for this data set was originally 0.119 in PDB entry 1gzm. The low *R*_merge_ for the hexagonal averaging presumably came about because the original scaling and merging removed much of the variation in the measurements in the trigonal data set.

The model refined in *P*6_4_ was obtained by superposing the two molecules from the *P*3_1_ asymmetric unit and retaining the solvent, detergent and additive molecules common to both. Translation of the model also was necessary to align the threefold screw axes in the two space groups.

The new model was initially refined with *REFMAC*5 (Murshudov *et al.*, 1997 ▶) in the *CCP*4 suite (Collaborative Computational Project, Number 4, 1994 ▶). *R*_free_ (Brünger, 1993 ▶) was calculated using 5% of the reflections. Weights on the geometric restraints were adjusted to produce r.m.s. deviations from ideality comparable to those reported in the original PDB file. NCS restraints were not applied even though they had been used in the original structure analysis. This was done in order to focus on the effects of imposing the higher space-group symmetry. σ_A_-weighted |*F*_o_| − |*F*_c_| and 2|*F*_o_| − |*F*_c_| electron-density maps (Read, 1986 ▶) were examined with *XtalView* (McRee, 1999 ▶) for manual adjustments of the models.

Refinement of the N2C/D282C mutant (PDB entry 2j4y) followed the same protocol except that the data set was re­indexed to make it comparable to that for the ground-state molecule. The index transformation applied was *h*(new) = *k*(old), *k*(new) = *h*(old), *l*(new) = −*l*(old). *R*_merge_ for the conversion from *P*3_1_ to *P*6_4_ was 0.113, which was again substantially lower than the original *R*_merge_ of 0.24 (PDB entry 2j4y). Also, the atom names and residue numbers for the hetero groups in the mutant model were changed to make them consistent with those of the ground-state structure.

A description of the refinements with *REFMAC*5 was submitted to *Acta Crystallographica* and the two referees pointed out that the data set for the mutant rhodopsin was twinned. The twinning server at UCLA (Padilla & Yeates, 2003 ▶) and the program *phenix.xtriage* (Zwart *et al.*, 2005 ▶; Adams *et al.*, 2002 ▶) indicated an approximate twinning fraction of 0.3 for the data set deposited for 2j4y and a twinning operation relating the *h*, *k*, *l* and −*k*, −*h*, −*l* reflections. A twinning fraction of 0.02 was obtained for the ground-state data set (1gzm). [Twinning was reported for the heavy-atom derivative used to solve this structure (Li *et al.*, 2004 ▶).]

The structures were refined with *phenix.refine* (Afonine *et al.*, 2005 ▶; Adams *et al.*, 2002 ▶) in space groups *P*3_1_ and *P*6_4_ with and without twinning corrections. Overall weighting of the restraints was adjusted to yield comparable r.m.s. deviations from ideal bond lengths in each refinement. *PRO­CHECK* (Laskowski *et al.*, 1993 ▶) and *MOLPROBITY* (Lovell *et al.*, 2003 ▶) were used to monitor and validate the structural models. Tables 1 ▶ and 2 ▶ contain refinement and validation information for the ground-state and mutant rhodopsins, respectively.

Refinement in the higher symmetry space group and inclusion of twinning led to a substantial improvement in the refinement of the mutant structure. Averaging of the additional replicated reflections by recognizing the crystallographic twofold operation parallel to the *z* axis must have improved the accuracy of the diffraction measurements. Recognition and appropriate treatment of the twinned reflections (reflections related by a twofold rotation perpendicular to the *z* axis) also improved the mathematical model for the diffraction pattern.

The refinement behavior and statistics for both molecules indicate that space group *P*6_4_ provides an appropriate description of these crystal structures. The protein molecules in each crystal structure are in identical environments (as far as the X-ray experiment is concerned) and not in two different environments as implied by the models in space group *P*3_1_. Coordinates and structure factors for these reinterpretations of the two structures have been deposited in the PDB and assigned identification codes 3c9l (ground state, *P*6_4_, untwinned) and 3c9m (mutant, *P*6_4_, twinned).

Refinement in the higher symmetry space group does not alter the fundamentals of the molecular packing in the non-isomorphous crystal structures. The two crystal structures still differ in the packing interactions between helices 5 in neighboring molecules that are now related by crystallographic symmetry operations rather than by noncrystallographic operations in space group *P*3_1_. The two modes of interaction between these helices are similar to those described by Melčák *et al.* (2007 ▶) in their discussion of the structure of Nup58/45. Nup58/45 is associated with nuclear pores and sliding interactions involving hydrophobic surfaces of antiparallel helices were suggested as being associated with regulation of the diameter of such pores. As pointed out by Standfuss *et al.* (2007 ▶), the different interactions in these two crystal structures are associated with different-sized solvent-filled channels. There is no known physiological function of these cavities, but they do provide a specific example of a structural feature that could have biological implications.

Another possible ramification of sliding interactions between molecules is that they could contribute to structural heterogeneity, complicating their crystallization. Alternate interactions between hydrophobic surfaces, whether formed by helices or β-sheets, could lead to a mixture of molecular packings inconsistent with the formation of a well ordered crystalline lattice. Identifying and controlling such interactions might aid in the crystallization of membrane proteins.

## Supplementary Material

PDB reference: rhodopsin, ground state, 3c9l, 3c9lsf

PDB reference: mutant, 3c9m, 3c9msf

## Figures and Tables

**Table 1:** Refinement statistics for ground-state rhodopsin

Space group	*P*3_1_	*P*3_1_	*P*3_1_	*P*6_4_	*P*6_4_
Refinement program	*CNS*	*phenix.refine*	*phenix.refine*	*phenix.refine*	*phenix.refine*
Resolution ()	2.65	2.65	2.65	2.65	2.65
*R* factor (overall)	0.207	0.189	0.190	0.188	0.189
*R*_free_	0.242	0.212	0.211	0.216	0.213
Twin fraction			0.03		0.02
No. of unique reflections	24704†	26063‡	26063	13785§	13785
No. of protein atoms	5206	5206	5206	2603	2603
No. of heteroatoms	546	546	546	265	265
No. of water molecules	40	40	40	20	20
Average *B* value, protein (^2^)	53.9	65.9	70.7	66.1	69.5
Average *B* value, nonprotein (^2^)	74.2	85.1	85.5	86.4	84.2
R.m.s. deviations					
Bond lengths ()	0.008	0.008	0.009	0.008	0.011
Bond angles ()	1.30	1.40	1.54	1.23	1.41
Ramachandran quality (*PROCHECK*), residues in					
Most favored regions (%)	89.6	89.5	89.6	89.5	89.5
Additional allowed regions (%)	8.1	8.8	8.8	9.1	9.1
Generously allowed regions (%)	2.3	1.6	1.6	1.4	1.4
Ramachandran quality (*MOLPROBITY*)					
Residues in favored regions (%)	94.5	93.8	94.2	93.8	94.5
Outliers (%)	1.5	1.4	1.2	1.2	1.2
PDB code	1gzm			3c9l	

† Number of reflections given in the PDB file.

‡ Number of reflections in the deposited structure-factor file.

§ Number of reflections after imposing hexagonal symmetry on the data set.

**Table 2:** Refinement statistics for the rhodopsin mutant (N2C/D282C)

Space group	*P*3_1_	*P*3_1_	*P*3_1_	*P*6_4_	*P*6_4_
Refinement program	*CNS*	*phenix.refine*	*phenix.refine*	*phenix.refine*	*phenix.refine*
Resolution ()	3.4	3.4	3.4	3.4	3.4
*R* factor (overall)	0.290	0.261	0.201	0.232	0.174
*R*_free_	0.330	0.274	0.216	0.300	0.219
Twin fraction			0.33		0.32
No. of unique reflections	13689†	13687‡	13687	7356§	7356
No. of protein atoms	5144¶	5178¶	5178	2589	2589
No. of heteroatoms	108¶	74¶	74	37	37
No. of water molecules	0	0	0	0	0
Average *B* value, protein (^2^)	50.2	74.4	81.8	64.0	72.0
Average *B* value, nonprotein (^2^)	51.5	77.4	84.5	70.1	76.9
R.m.s. deviations					
Bond lengths ()	0.014	0.014	0.019	0.010	0.012
Bond angles ()	1.66	1.52	1.70	1.47	1.50
Ramachandran quality (*PROCHECK*), residues in					
Most favored regions (%)	71.7	70.9	70.0	74.0	73.7
Additional allowed regions (%)	25.6	26.7	27.5	23.2	23.5
Generously allowed regions (%)	1.6	1.4	1.2	1.8	2.1
Disallowed regions (%)	1.1	1.2	1.1	1.1	0.7
Ramachandran quality (*MOLPROBITY*)					
Residues in favored regions (%)	79.0	78.9	78.9	78.2	78.4
Outliers (%)	3.9	4.2	4.0	4.0	3.7
PDB code	2j4y				3c9m

† Number of reflections given in the PDB file.

‡ Number of reflections in the deposited structure-factor file.

§ Number of reflections after imposing hexagonal symmetry on the data set.

¶ Some residues in the original structure determination were classified as heteroatoms for application of restraints. The total number of atoms is the same for all refinements in space group *P*3_1_.
